# Comparative analysis of copy number detection by whole-genome BAC and oligonucleotide array CGH

**DOI:** 10.1186/1755-8166-3-11

**Published:** 2010-06-29

**Authors:** Nicholas J Neill, Beth S Torchia, Bassem A Bejjani, Lisa G Shaffer, Blake C Ballif

**Affiliations:** 1 Signature Genomic Laboratories, Spokane, WA, USA

## Abstract

**Background:**

Microarray-based comparative genomic hybridization (aCGH) is a powerful diagnostic tool for the detection of DNA copy number gains and losses associated with chromosome abnormalities, many of which are below the resolution of conventional chromosome analysis. It has been presumed that whole-genome oligonucleotide (oligo) arrays identify more clinically significant copy-number abnormalities than whole-genome bacterial artificial chromosome (BAC) arrays, yet this has not been systematically studied in a clinical diagnostic setting.

**Results:**

To determine the difference in detection rate between similarly designed BAC and oligo arrays, we developed whole-genome BAC and oligonucleotide microarrays and validated them in a side-by-side comparison of 466 consecutive clinical specimens submitted to our laboratory for aCGH. Of the 466 cases studied, 67 (14.3%) had a copy-number imbalance of potential clinical significance detectable by the whole-genome BAC array, and 73 (15.6%) had a copy-number imbalance of potential clinical significance detectable by the whole-genome oligo array. However, because both platforms identified copy number variants of unclear clinical significance, we designed a systematic method for the interpretation of copy number alterations and tested an additional 3,443 cases by BAC array and 3,096 cases by oligo array. Of those cases tested on the BAC array, 17.6% were found to have a copy-number abnormality of potential clinical significance, whereas the detection rate increased to 22.5% for the cases tested by oligo array. In addition, we validated the oligo array for detection of mosaicism and found that it could routinely detect mosaicism at levels of 30% and greater.

**Conclusions:**

Although BAC arrays have faster turnaround times, the increased detection rate of oligo arrays makes them attractive for clinical cytogenetic testing.

## Introduction

Molecular cytogenetic techniques such as array-based comparative genomic hybridization (aCGH) have revolutionized cytogenetic diagnostics and, in turn, the clinical management of patients with developmental delays and multiple congenital anomalies [1,2]. These rapid, high-resolution, and highly accurate techniques have identified numerous previously unrecognized chromosomal syndromes [3-8], refined critical regions for established genetic defects [9], and broadened our view of the "normal" diploid genome [10]. In addition, aCGH has given the clinician a greater appreciation of variability in the clinical presentation of many well-described conditions [11,12] and allowed for the discovery of new conditions with relatively mild phenotypes [13,14]. Furthermore, the application of aCGH has created a paradigm shift in genetics that has moved the description and discovery of genetic conditions from the "phenotype-first" approach, in which patients exhibiting similar clinical features are identified prior to the discovery of an underlying etiology, to a "genotype-first" approach, in which a collection of individuals with similar copy-number imbalances can be examined for common clinical features [15].

Originally, targeted microarrays constructed from bacterial artificial chromosomes (BAC) were developed for the clinical laboratory because of their ability to clearly identify copy number changes in discrete regions of the human genome known to play a role in genetic disease [16]. This "less is more" idea prevailed in the early years of clinical aCGH because the technology was new and proof of principle was required before it could be adopted for more widespread diagnostic use. Furthermore, the identification of copy number alterations of unclear clinical significance was considered undesirable to the diagnostician, the ordering physician, and the patient's family. Recently, the coverage of microarrays has expanded to include more comprehensive coverage of the human genome, leading many to suggest that whole-genome BAC or oligo arrays are the next step in the continued improvement in the detection rate of cytogenetic abnormalities.

It has been presumed that whole-genome oligonucleotide arrays, because they have higher resolutions, would detect more copy number aberrations than whole-genome BAC arrays. However, to our knowledge, there has not been a systematic comparison of these two whole-genome copy number screening technologies in a clinical diagnostic environment. Therefore, to determine which platform is most effective in identifying clinically significant DNA copy number alterations, we designed a whole-genome BAC array and a whole-genome oligo array and compared the results in a blinded study of 466 clinical diagnostic specimens. In addition, we prospectively evaluated 3,443 patients by the whole-genome BAC array and 3,096 patients by the whole-genome oligo array and compared the detection rates of clinically significant abnormalities and those of unclear clinical significance. Finally, we validated our oligo array with 48 cases to determine the level of mosaicism that can be reliably detected and compared that level to our previously published cases analyzed using the BAC array.

## Materials and methods

### Whole-genome BAC array design and aCGH

We constructed a whole-genome BAC array designed for clinical diagnostic use using >4,600 BAC clones. All clones were validated by FISH prior to inclusion on the array using previously described validation procedures [16]. Contigs of 3-6 overlapping clones were selected to cover 1,543 genetic loci, including >150 known microdeletion/microduplication syndromes and increased density of coverage in the 5-10 Mb surrounding the subtelomeric and pericentromeric regions of the genome. In addition, we placed contigs to cover >500 functionally significant genes such as transcription factors and other genes known to play important roles in development. This coverage also includes genome-wide representation with at least one contig in nearly every chromosomal band at the resolution of an 850-band karyotype. The mean gap size for the whole-genome BAC array is ~1.6 Mb. Microarray manufacturing and aCGH analysis using the whole-genome BAC array were performed as previously described [13]. BAC arrays were analyzed after a dye-swap, two-experiment analysis [16], using sex-mismatched controls. Results were then displayed using custom BAC aCGH analysis software (Genoglyphix™; Signature Genomic Laboratories, Spokane, WA).

### Whole-genome Oligonucleotide Array Design and aCGH

Oligonucleotide-based microarray analysis was performed using a custom-designed, 105K-feature whole-genome microarray manufactured by Agilent Technologies (Santa Clara, CA) with one probe every 10 kb in regions of interest--microdeletion/microduplication syndromes, the pericentromeric regions, subtelomeres and genes involved in important developmental pathways--for an average of 50 oligos per clinical locus. In addition, to achieve backbone coverage, we placed a probe, on average, every 35 kb throughout the rest of the genome between the regions of interest. Genomic DNA labeling was performed as described for BAC arrays, whereas array hybridization and washing were performed as specified by the manufacturer (Agilent Technologies). A dye swap was not performed for the oligo arrays, and sex-matched controls were used. Arrays were scanned and analyzed as previously described [17]. Results of aberration calls consisting of five or more consecutive oligos were then displayed using custom oligonucleotide aCGH analysis software (Genoglyphix™; Signature Genomic Laboratories). The use of five consecutive oligos achieved a resolution of 40 kb in the regions of interest and a resolution of 140 kb in the backbone.

### Decision Algorithm for Clinically Significant Copy Number Reporting

We developed a decision algorithm for classifying clinically significant copy number alterations, alterations of unclear clinical significance, and alterations of no currently known clinical significance. Alterations that were associated with established chromosomal syndromes, were large and affected a significant amount of gene content, or were part of a complex rearrangement such as an unbalanced translocation, insertion, or marker chromosome were characterized as clinically significant. Alterations with unclear clinical significance were most commonly those which were not currently associated with a syndrome but which affected gene content which may have contributed to the patient's phenotype and those which could not be precisely refined by the BAC array. Alterations were considered to have no known clinical significance if they were small, affected minimal gene content, and/or were present in regions where common copy-number variation was known to occur in the general population. Signature's own Genoglyphix Chromosome Aberration Database (GCAD) was used as a reference to assist in the interpretation of each alteration. GCAD is a database of >11,000 chromosomal abnormalities identified in >9,500 patients out of >40,000 patients evaluated by our laboratory and contains detailed statistics of each observed alteration (breakpoint coordinates, size, gene content, etc.) as well as clinical information pertaining to patient referral.

### Fluorescence in situ Hybridization (FISH)

When possible, all copy number alterations detected by microarray analysis were visualized by interphase and/or metaphase FISH using a BAC probe located within the region of gain or loss. FISH was performed as previously described [18].

### Patient Clinical Testing

To validate the custom-designed 105k oligo array compared to the whole-genome BAC array, 466 cases were run side-by-side in a platform comparison study. In each case, the clinically validated BAC array results were used for interpretation and reporting. Specimens with known chromosome abnormalities, parental specimens, and prenatal cases were excluded from the analysis.

In addition, we conducted a prospective study of 3,443 consecutive BAC microarray analyses and 3,096 consecutive oligo microarray analyses in our clinical laboratory. The array platform used for testing in each case was chosen by the referring physician at the time of sample submission to our clinical diagnostic laboratory. Cases with previously known chromosomal abnormalities, parental samples, and prenatal specimens were again excluded from the data collection.

### Mosaicism Assessment

The ability of the oligo platform to detect mosaicism was assessed on 48 patients previously known to carry mosaic abnormalities at levels as low as 5%. The alterations studied included a variety of interstitial, terminal, and whole-chromosome copy-number abnormalities, as well as marker chromosomes. The mosaic alteration in each patient was initially assessed by BAC array and the level of mosaicism determined by interphase FISH analysis when possible. In a separate experiment, mosaicism was assessed using a dilution of cells from a male with trisomy 21 with normal male control cells, as previously described [19]. After FISH verification of the dilutions, DNA was extracted from the diluted cells, labeled and hybridized to the custom-designed oligo array as described above.

## Results

### Platform comparison study

From the 466 cases analyzed by the BAC array, using the previously described algorithm, we excluded 347 cases that only had aberrations located within regions that contained no genes and/or aberrations that had been established to be normal population variants by Signature Genomic Laboratories or identified in the Toronto Database of Genomic Variants (DGV, http://projects.tcag.ca/variation/). After these cases were excluded, 138 copy number alterations in 119 cases (25.5% of the original 466 cases) remained that required FISH analysis. These aberrations included subtelomeric and pericentromeric gains for which FISH was required to exclude an unbalanced translocation or a marker chromosome. After FISH was performed, 60 aberrations in 52 cases were classified as normal variants because marker chromosomes and derivative chromosomes were not identified and because these alterations were located within regions where common copy number variation is known to occur. Thus, alterations of potential clinical significance according to our algorithm were identified in 67 cases, a detection rate of 14.4%. Of these cases, 56 (12.0%) were considered to contain clinically significant copy number alterations (Table 1), and 11 (2.4%) were considered to contain copy number variants of unclear clinical significance for which parental analyses were recommended to further clarify the abnormality (Table 2). aCGH and FISH analysis performed on parental samples revealed that six alterations of unclear significance were inherited from a carrier parent and one was a *de novo *event in the proband. The origin of the other four unclear alterations could not be determined.

**Table 1:** Cases with Alterations of Clinical Significance Identified by BAC and Oligo aCGH.

Pt. #	Chr	Band	**Start pos**.	**End pos**.	Gain/Loss	Size	# of BACs	# of Oligos
**21637**	chr16	p11.2	29,563,985	30,066,187	Loss	502,202	5	46

**21992**	chr16	p11.2	29,563,985	30,066,187	Loss	502,202	5	46

**22013**	chr16	p11.2	29,563,985	30,066,187	Loss	502,202	5	46

**21993**	chr10	q25.2q25.3	114,306,207	114,925,368	Loss	619,161	3	36

**21756**	chr2	q35	219,418,281	220,060,969	Loss	642,688	2	43

**22002**	chr2	q37.3	239,664,393	240,400,008	Loss	735,615	3	82

**22334**	chr10	q25.2q25.3	114,024,053	115,677,301	Gain	1,653,248	3	53

**21688**	chr16	p13.11p12.3	15,056,257	16,742,812	Gain	1,686,555	3	61

**21667**	chr17	p12	14,052,297	15,742,271	Gain	1,689,974	3	67
**21896**	chr4	q35.2	189,407,487	191,133,809	Loss	1,726,322	8	147

**22269**	chr2	q12.3q13	107,945,041	109,784,684	Loss	1,839,643	6	102

**21640**	chr19	q13.42	59,272,450	61,239,237	Gain	1,966,787	8	135

**22117**	chr9	q33.1	118,991,777	121,063,590	Gain	2,071,813	3	67

**22066**	chr8	p12p11.21	38,303,146	40,515,492	Gain	2,212,346	6	104

**21786**	chr1	q21.1	144,973,942	147,421,814	Gain	2,447,872	5	63

**22237**	chr1	q21.1	144,973,942	147,421,814	Gain	2,447,872	5	63

**22310**	chr22	q11.21	17,299,742	19,770,655	Loss	2,470,913	13	205

**22050**	chr22	q11.21	17,007,819	19,770,655	Loss	2,762,836	13	208

**21936**	chr1	q21.1	144,973,942	147,966,185	Gain	2,992,243	5	65

**21719**	chr2	q31.1	169,823,689	172,870,083	Loss	3,046,394	8	119

**22128**	chr4	q34.3	179,065,989	182,435,119	Loss	3,369,130	3	91

**22006**	chr17	p11.2	16,723,071	20,145,604	Loss	3,422,533	18	311

**22174**	chr1	q41q42.12	221,260,860	224,709,317	Loss	3,448,457	9	178

**21971**	chr22	q11.23q12.2	24,025,269	28,008,109	Loss	3,982,840	3	111

**22073**	chr15	q11.2q13.1	21,208,177	26,194,049	Loss	4,985,872	11	281

**21687**	chr16	q12.2q21	51,912,655	57,173,018	Loss	5,260,363	15	261

**21975**	chr5	p15.2p14.3	13,514,464	18,988,928	Loss	5,474,464	3	122

**21555**	chr7	p22.3p22.1	153,644	6,230,285	Gain	6,076,641	32	434

**21547**	chr2	q24.3q31.1	168,702,606	174,842,496	Loss	6,139,890	11	226

**21755**	chr11	p12p11.2	37,540,680	43,940,573	Loss	6,399,893	14	298

**21761**	chr3	p14.1p12.3	70,738,914	77,275,908	Loss	6,536,994	6	151

**22151**	chr15	q11.2q13.1	18,809,804	26,194,049	Gain	7,384,245	14	331

**22322**	chr8	p21.3p12	22,954,212	30,630,828	Loss	7,676,616	9	224

**21889**	chr2	q33.1q34	202,901,021	211,366,732	Gain	8,465,711	13	281

**21723**	chr5	q23.1q23.3	121,487,477	130,306,377	Loss	8,818,900	4	204

**22337**	chr1	p36.22p36.13	9,476,880	19,436,653	Loss	9,959,773	21	436

**21795**	chr1	p34.2p32.3	41,201,837	55,191,500	Gain	13,989,663	17	440

**20986**	chr12	p13.33p12.3	84,918	17,505,135	Gain	17,420,217	46	800

**21957**	chr11	q23.3q25	116,478,434	134,419,382	Gain	17,940,948	40	784

**21596**	chr1	q25.1q32.1	173,519,967	203,663,817	Gain	30,143,850	25	814

**22055**	chr3	p14.1p13	71,164,161	71,958,845	Loss	794,684	3	46

21566*	chr17	p13.2p13.1	6,081,457	6,904,679	Loss	823,222	3	14

**21558**	chr22	q13.33	48,567,185	49,517,230	Loss	950,045	6	54

**21770**	chr8	p23.3	202,505	1,411,517	Loss	1,209,012	5	96

**22254**	chr8	p23.2	2,604,280	3,966,809	Loss	1,362,529	9	140

**21937**	chr7	q11.23	72,404,049	73,771,409	Loss	1,367,360	10	158

**21710**	chr15	q13.2q13.3	28,741,818	30,186,356	Loss	1,444,538	3	64

**21722**	chr15	q13.2q13.3	28,741,818	30,226,376	Loss	1,484,558	3	65

**21739**	chr15	q13.2q13.3	28,741,818	30,226,376	Loss	1,484,558	3	65

**21787**	chr15	q13.2q13.3	28,741,818	30,226,376	Loss	1,484,558	3	65

21897*	chr5	p15.2	8,511,592	9,888,817	Gain	1,377,225	34	29

**21884**	chrX	q28	152,676,750	153,059,428	Gain	382,678	3	44

**21592**	chrX	p22.33q28	701	154,888,083	Gain	154,887,382	325	6888

**22087**	chrY	p11.32	262,578	57,715,879	Gain	57,453,301	49	49

**22285**	chrX	p21.1	31,759,551	31,830,811	Loss	71,260	2	11

**22244**	chr22	q13.33	49,342,961	49,514,486	Loss	171,525	2	56

*additional alterations identified by oligonucleotide aCGH.								

**Table 2:** Cases with Alterations of Unclear Significance Identified by BAC and Oligo aCGH.

Pt. #	Chr	Band	Start pos.	End pos.	Gain/Loss	Size	# of BACs	# of Oligos	Inheritance
**22365**	chr9	p23	9,881,385	9,984,838	Loss	103,453	2	15	Paternal

**21702**	chr16	p12.2	21,486,897	21,641,890	Loss	154,993	2	16	Paternal

**21883**	chr16	p12.1	21,974,396	22,338,234	Gain	363,838	3	49	Maternal

**21860**	chr16	p12.1	21,907,270	22,338,234	Loss	430,964	3	50	Unknown

**22009**	chr22	q11.21	19,069,125	19,770,655	Loss	701,530	4	70	Paternal

**22102**	chr16	p13.11	14,981,044	16,166,985	Gain	1,185,941	3	60	Maternal

**22246**	chr10	p11.22	31,591,310	32,792,762	Loss	1,201,452	2	40	Unknown

**21893**	chrX	p11.32	45,930,652	46,382,140	Gain	451,488	3	34	Maternal

**22273**	chrX	q28	153,355,101	154,317,591	Gain	962,490	5	46	Unknown

**10245**	chr3	p14.1	67,727,841	69,101,769	Loss	1,373,928	2	25	Unknown

**22348**	chr13	q22.2	74,989,699	75,378,640	Loss	388,941	3	51	De novo

Using the oligo array, we identified 1,337 copy number variations among the same 466 cases. Using the algorithm previously described, we excluded 1,172 aberrations that were located within regions that had no gene content or those that were common copy number variants. After these exclusions were made, 165 aberrations in 138 cases (29.6%) remained that required FISH analysis. After FISH analysis was performed, aberrations of potential clinical significance were identified in 73 cases, a detection rate of 15.7%. Of these, the same 56 (12.0%) cases that were identified by the BAC platform were considered to contain clinically significant alterations (Table 1) and 17 (3.7%) were determined to contain copy number variants of unclear clinical significance.

Table 3 shows the six cases for which aberrations of unclear clinical significance were identified by the oligo array but not by the BAC array. In all six cases, the aberrations either fell within the gaps in the BAC array coverage or were only partially covered by one or more BACs. The average size of the alterations that were not detected by the BAC array was 1.12 Mb (range: 289 kb - 1.42 Mb).

**Table 3:** Cases with Alterations of Unclear Significance Detected by Oligo Array but not Identified by BAC Array.

Pt. #	Chr	Band	Start pos.	End pos.	Gain/Loss	Size	# of Oligos
**9756**	chr2	p25.1	11,097,126	12,515,559	Loss	1,418,433	19

**9886**	chr1	q42.12	222,702,622	223,461,255	Loss	758,633	16

**10141**	chr2	q32.3q33.1	196,729,308	197,880,950	Loss	1,151,642	30

**10114**	chr15	q26.3	97,299,441	97,745,782	Gain	446,341	16

**10292**	chr7	p14.3	33,202,932	33,492,136	Loss	289,204	5

**10019**	chr2	p16.3	51,079,474	51,993,245	Gain	913,771	13

**Figure 1:**
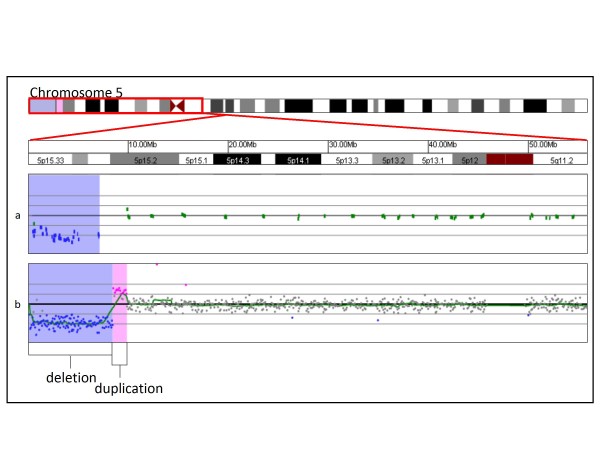
**Identification by oligonucleotide microarray of additional complexity missed by BAC microarray**. **(A) **BAC microarray results showing a single-copy loss of 34 BAC clones from the terminus of 5p, approximately 6.8 Mb in size (chr5: 387,034-7,150,950, based on UCSC 2006 hg 18 assembly). Probes are ordered on the x axis according to physical mapping positions, with the p-arm probes to the left and q-arm probes to the right. **(B) **shows oligonucleotide microarray results of the terminal deletion shown in (A) in addition to single-copy gain of 29 probes from 5p, approximately 1.38 Mb in size (chr5: 8,511,592-9,888,817, based on UCSC 2006 hg 18 assembly). Probes are ordered as in the BAC array. Regions shaded in blue represent deletions detected by microarray, whereas duplications are shaded in pink.

In two cases, the oligo microarray identified additional complexity that was not recognized by the BAC array. In patient 21566, the BAC array identified one interstitial deletion of 17p13.2p13.1, whereas oligo array analysis identified that deletion and an additional interstitial deletion in the same band (data not shown). In patient 21897, the BAC array identified a 6.8 Mb terminal deletion of 5p, whereas oligo array analysis identified that deletion and a 1.4 Mb duplication proximal to the deleted region (Figure 1).

### Prospective Diagnostic Comparison

Of the 3,443 diagnostic specimens analyzed using our whole-genome BAC array, 605 (17.6%) had copy number alterations. Using the previously described algorithm, 365 (10.6%) had abnormalities that were classified as clinically significant, whereas 240 (6.9%) had copy number variants of unclear clinical significance.

Of the 3,096 diagnostic specimens analyzed using our whole-genome oligo array during the same time period, 698 (22.5%) had copy number alterations. Using the previously described algorithm, 477 (15.9%) of these cases were determined to contain alterations considered to be clinically significant and 221 (7.0%) were determined to contain copy number variants of unclear clinical significance (Table 4).

**Table 4:** Summary of the Prospective Diagnostic Comparison.

	BAC	Oligo
**Total**	3,443	3,096

**Abnormal**	605 (17.6%)	698 (22.5%)

**Significant**	365 (10.6%)	477 (15.4%)

**Unclear**	240 (7.0%)	221 (7.1%)

**Mosaic**	16 (0.5%)	12 (0.4%)

The increased number of cases with clinically significant alterations detected by the oligo array was found to be statistically significant using a Fisher's Exact Test (OR = 1.5359, p < .0001). The increased number of cases with alterations of unclear significance detected by the oligo array was not statistically significant (OR = 1.0259, p = 0.8090).

### Mosaicism Assessment

**Figure 2:**
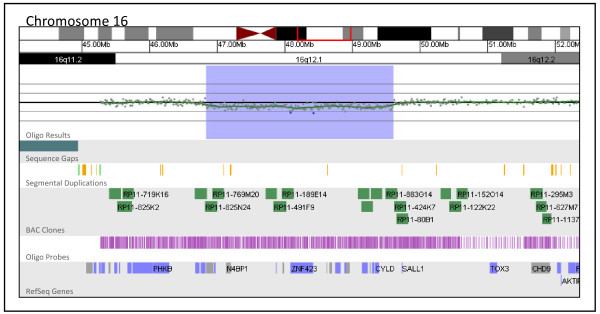
**Oligonucleotide microarray analysis of a mosaic 16q12.1 deletion (shaded blue region)**. The zoomed-in microarray plot shows a single-copy loss of 289 probes from 16q12.1, approximately 2.77 Mb in size (chr16: 46,837,260-49,605,054, based on UCSC 2006 hg 18 assembly). Probes are ordered on the x axis according to physical mapping positions, with the most proximal 16q11.2 probes to the left and the most distal 16q12.2 probes to the right.

All but three of the 48 previously known mosaic alterations were detected by the oligo array. FISH analysis estimated that the proportion of uncultured cells carrying the alteration was 24% in the first case, while the proportion in cultured cells was 6%. In the second case, 5% of cells were found to carry the alteration by FISH (data not shown). The proportion of cells carrying the alteration in the third case could not be determined because FISH confirmation was not possible on the sample received by our laboratory. Certain alterations, such as tetrasomy 12p, were successfully detected in proportions of cells as low as 10% by the oligo array, although this low threshold of detection was facilitated by the tetrasomic nature of the rearrangement; the 4:2 ratio of patient to control DNA in this case was more readily detected than the 3:2 ratio typically associated with duplications. Figure 2 shows a 2.77 Mb interstitial deletion at 16q12.1 present in 23% of cultured metaphase cells that was detected by the oligo array.

**Figure 3:**
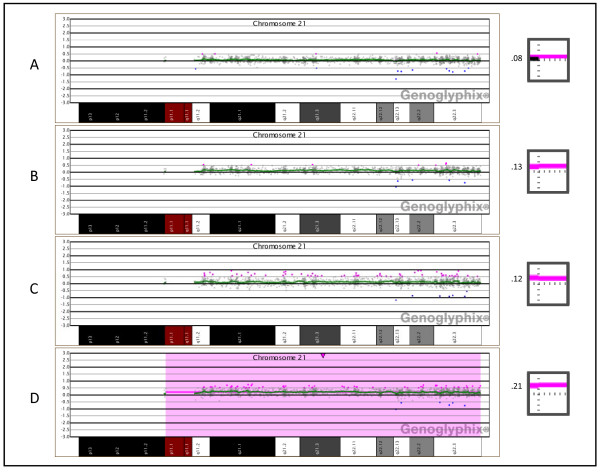
**Oligonucleotide microarray analysis of artificially derived mosaic trisomy 21 samples**. **(A) **10% trisomy 21 showing a very subtle copy-number gain for all clones on chromosome 21. The profile was generated using DNA extracted from a mixture of blood which contained 10% WBCs from a trisomy 21 subject and 90% WBCs from a normal male individual. **(B) **15% trisomy 21, generated as in (A), showing a very subtle copy-number gain for all clones on chromosome 21. **(C) **20% trisomy 21, generated as in (A) showing a subtle copy-number gain for all clones on chromosome 21. **(D) **30% trisomy 21, generated as in (A), showing a clear copy-number gain for all clones on chromosome 21. The inset images to the right of each array plot show the average log_2 _ratio of all probes mapping to chromosome 21, with the horizontal dotted line representing a log_2 _ratio of zero and the vertical dotted line representing the centromere. A pink bar plotted above the horizontal line represents a copy-number gain of all probes on chromosome 21. To the left of each inset image is the average log_2 _ratio at the specified proportion of trisomic cells.

In the dilution series of trisomy 21 cells, shifts in the aCGH data were distinguishable down to levels as low as 10%, but could only be readily detected at a level of 30% or greater (Figure 3). As the proportion of trisomy 21 cells was increased from 10% to 30%, the average log_2 _ratio of chromosome 21 increased from 0.08 to 0.21. During the prospective diagnostic comparison, 16 cases analyzed using the BAC array contained mosaic alterations, whereas only 12 mosaic cases were identified using the oligo array (Table 5). The increased number of mosaic abnormalities detected by the BAC array was determined to be not statistically significant (OR = 1.1999, p = 0.7066).

**Table 5:** Mosaic Alterations Detected in the Prospective Diagnostic Comparison.

Pt. #	Proportion (%)	Classification
**BAC**		

**25885**	10	45,X

**25838**	10	47,XX,i(12)(p10)

**27745**	10	46,XY,trp(12)(p13.33p10)

**26912**	18	47,XY,i(8)(p10)

**26358**	20	46,XX,dup(2)(p14p11.2)

**26880**	24	47,XX,+9

**23302**	27	47,XX,+der(9)(p21.2q11)

**23919**	46	47,XY,+der(12)(p13.33q11)

**26127**	53	46,XY,del(18)(q22.3q23)

**26750**	57	47,XY,+der(8)(p11.22q11)

**26894**	60	45,X

**24887**	70	46,XX,der(14)dup(14)(q32.13q32.2)del(14)(q32.3q32.33)

**23159**	70	47,XYY

**23155**	77	46,XX,idic(18)(q21.33)

**23215**	87	47,XX,+21

**25862**	93	48,XX,+der(13)(pterq12.12),+der(20)(p11.21q11)

**Oligo**		

**27978**	21	48,XY,+der(13)(pterq12.11),+der(?)(?::Xp22.31->Xp22.31::Xp22.2->Xp22.12::?->cen->?)

**32047**	27	47,XXY

**32374**	27	46,XX,r(X)(p11.1q21.1)

**32875**	33	47,XY,+inv dup(22)(q11.21)

**29361**	53	47,XX,+der(11)(p11.2q11)

**31439**	63	47,XY,+der(12)(:p13.33::p13.31->p13.2::p11.23::p11.22->p11.21::?->12cen->?::p11.21->p11.22::p11.23::p13.2->::p13.31::p13.33:)

**31633**	63	48,XX,+der(4)(p13q12),+der(13)(pterq12.11)

**30028**	77	46,XY,del(7)(q22.1q22.3),del(12)(q21.31q22)

**31336**	80	46,XX,idic(X)(q21.1)

**27105**	90	47,XX,+der(13)(pterq12.12)

**29786**	90	46,X,+der(X)(p11.21q11.1)

**30218**	93	47,XY,+der(17)(p11q11.2)

## Discussion

**Figure 4:**
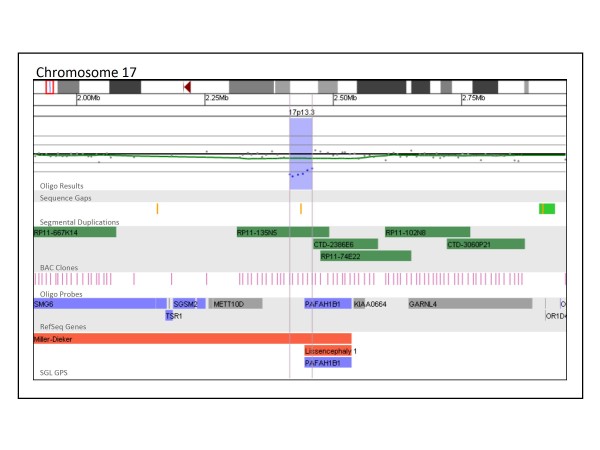
**Oligonucleotide microarray characterization of an interstitial deletion at 17p13.3**. The zoomed-in microarray plot shows a single-copy loss of six probes from the short arm of chromosome 17 at 17p13.3, approximately 44.0 kb in size (chr17: 2,415,074-2,459,051, based on UCSC 2006 hg 18 assembly). Probes are ordered on the x axis according to physical mapping positions, with the most distal 17p13.3 probes to the left and the most proximal 17p13.3 probes to the right. Below is a schematic of the deletion region. The deletion disrupts the *PAFAH1B1/LIS1 *gene.

**Figure 5:**
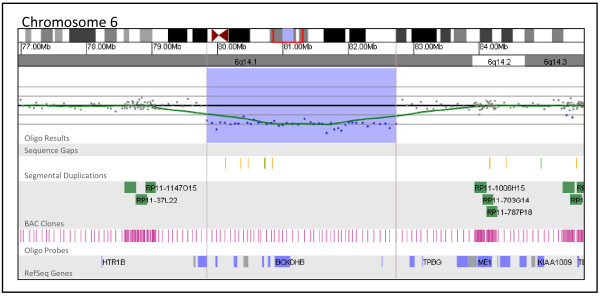
**Oligonucleotide microarray characterization of an interstitial deletion at 6q14.1**. The zoomed-in microarray plot shows a single-copy loss of 43 oligonucleotide probes from the long arm of chromosome 6 at 6q14.1, approximately 2.9 Mb in size (chr6: 79,838,518-82,730,466, based on UCSC 2006 hg 18 assembly). Probes are ordered on the x axis according to physical mapping positions, with the most proximal 6q14.1 probes to the left and the most distal 6q14.1 probes to the right. Below is a schematic of the deletion region. Blue and gray boxes represent genes in the deletion region.

BAC and oligo array platforms each have unique advantages and disadvantages in a diagnostic setting; these may include turnaround times, genomic coverage, and costs. One of the most important characteristics of each platform is the detection rate of clinically significant alterations. Our results demonstrate that our whole-genome oligo array was able to detect such alterations in 15.4% of patients tested, compared to the BAC array detection rate of 10.6%, a statistically significant difference (Fisher's Exact Test, p < 0.0001). The alterations that constitute the 4.8% difference in detection rate between the BAC and oligo arrays are either too small to be detected by the BAC array but are not below the resolution of the oligo platform (Figure 4) or fall within gaps in the BAC array coverage (Figure 5). Figure 4 shows a 44 kb deletion of 17p13.3 detected in a patient referred to our laboratory for convulsions. This deletion encompasses the first exon of *PAFAH1B1 *(*LIS1*). While it is not known whether this deletion results in a null allele or simply a truncated gene product, hemizygous deletions and mutations of this gene are found in patients with isolated lissencephaly type 1 (OMIM 607432) and have been linked to epileptic seizures and convulsions [20,21]. Although RP11-135N5 provides coverage of this region on the BAC array, FISH analysis using this clone could not confirm the deletion in any cells because of the deletion's small size compared to the FISH probe used. Thus, this clinically significant deletion could only have been reliably detected using the oligo platform. Although oligo-based aCGH has the power to detect alterations smaller than the size of a BAC probe, BAC-based aCGH has an advantage in that the analysis makes evident the appropriate probe to be used for FISH confirmation. In addition, this probe is usually readily available because of its inclusion on the microarray platform and will have a high rate of successful confirmation. When oligonucleotide-based aCGH is performed, BAC probes must be specifically selected for the FISH confirmation of each small abnormality that is detected. Once a probe has been selected, it must also be specially prepared or ordered before FISH can be performed. This process increases both the time it takes to perform FISH confirmation of oligo aCGH results and the cost associated with the analysis.

Figure 5 shows a 2.9 Mb deletion of 6q14.1 detected in a patient referred to our laboratory for developmental delay and dysmorphic features. This deletion encompasses eight genes: *PHIP*, *HMGN3*, *LCA5*, *SH3BGRL2*, *ELOVL4*, *TTK*, *BCKDHB*, two of which are known to be associated with human disease [22-24]. Although this 2.9 Mb deletion is likely to be clinically significant, it lies within a gap in the coverage of our BAC array and could only be detected using the oligo platform because of its more uniform backbone coverage.

**Figure 6:**
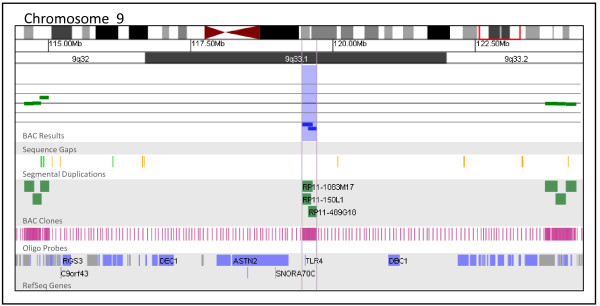
**BAC microarray characterization of a 9q33.1 deletion**. The zoomed-in microarray plot shows a single-copy loss of three BAC clones from the long arm of chromosome 9 at 9q33.1, approximately 262 kb in size (chr9: 119,452,279-119,714,054 based on UCSC 2006 hg 18 assembly). The nearest distal clone on chromosome 9 that is not deleted is RP11-977E8 and is approximately 4.0 Mb away from the deleted region. The nearest proximal clone on chromosome 9 that is not deleted is RP11-999I23 and is approximately 4.4 Mb away from the deleted region. Probes are ordered on the x axis according to physical mapping positions, with proximal 9q32 clones to the left and distal 9q33.2 clones to the right. Below is a schematic of the deletion region. Vertical blue lines represent the minimum size of this alteration, which encompasses one gene, *TLR4*.

The detection rate of alterations of unclear clinical significance is also a concern during the selection of a microarray platform in a clinical diagnostic setting. Our data suggest that both the oligo and BAC platforms detect similar numbers of abnormalities of unclear significance (7.0% by BAC and 7.1% by oligo), although the circumstances leading to an unclear clinical interpretation may vary between the platforms. On the BAC platform, unclear results are often associated with gaps in coverage which prevent the precise determination of the breakpoints and gene content of an abnormal region. This lack of information prohibits definitive interpretation of the clinical significance of the alteration. Figure 6 presents a 262 kb deletion of 9q33.1 detected by BAC array in a patient referred for developmental delay, dysmorphic features, and multiple congenital anomalies. The boundaries of this alteration as defined by BAC array include only one gene, *TLR4 *[25]. However, gaps in BAC coverage on both sides of the alteration span 4.5 Mb proximally and 4.0 Mb distally. As a result of these coverage gaps, this alteration, though estimated to be just 262 kb, may be as large as 8.7 Mb and include up to 48 additional genes. The design of BAC arrays with dense clone coverage is possible; however, probe density is limited by the availability of BAC clones and the presence of potentially interfering genomic architecture such as segmental duplications. In addition, BAC-based microarrays will not reliably detect abnormalities smaller than the size of an individual probe--80-200 kb, on average, for BAC clones.

**Figure 7:**
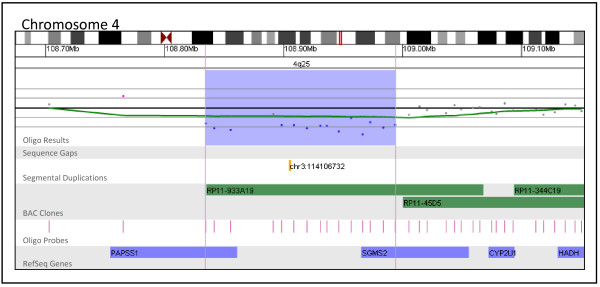
**Oligonucleotide microarray characterization of an interstitial deletion at 4q25**. The zoomed-in microarray plots shows a single-copy loss of 15 oligonucleotide probes from the long arm of chromosome 4 at 4q25, approximately 159.6 kb in size (chr4: 108,834,399-108,994,048, based on UCSC 2006 hg 18 assembly). Probes are ordered on the x axis according to physical mapping positions, with proximal 4q25 clones to the left and distal 4q25 clones to the right. Below is a schematic of the deletion region. The deletion disrupts the *PAPSS1 *and *SGMS2 *genes, represented by blue boxes.

Although gaps in coverage and limited breakpoint-resolving power are primarily a concern for BAC platforms, both oligo and BAC platforms produce results that are unclear because a lack of published evidence prevents a conclusive association between the gene content of an alteration and the clinical features of the patient from being made. Figure 7 presents a 160 kb deletion of 4q25 detected by oligo array in a patient referred for developmental delay. Follow-up analysis performed on this patient's parents revealed that this alteration was *de novo *in origin. This alteration deletes two genes, *PAPSS1 *and *SGMS2*. While mutations or alterations of these genes have not been associated with disease in humans, it has been shown that PAPSS1 plays a key role in post-translational modification and SGMS2 mediates the production of sphingomyelin [26,27]. Thus, although the gene content and inheritance pattern of this deletion suggest a causative role in the patient's clinical features, a lack of published information linking the genes affected by this alteration with a distinct phenotype prevents a clear interpretation from being made based on only aCGH results. This type of unclear result, although more prominent with oligo platforms (4.2% by BAC vs. 7.1% by oligo), is an element of all aCGH analysis regardless of platform and accentuates the need for databases containing aCGH results in combination with phenotypic information. Although the number of characterized genetic disorders and genomic regions is rapidly increasing, the clinical consequences of alterations involving much of the genome still remain unclear.

The increase in the number of copy number alterations identified by higher-resolution whole-genome arrays underscores the need for a variety of tools to facilitate the interpretation of array results in a clinical diagnostic setting. We propose the use of an algorithm such as the one outlined here in conjunction with databases of normal population variants, clinically significant alterations, and those of unclear significance. Although such databases can provide invaluable context for the analysis of aCGH data, care must be taken by the diagnostician when comparing their data to pre-existing databases of copy-number variations. For example, data in the DGV are pooled from a variety of sources, platforms, and populations using a variety of different controls and without independent verification, and thus may not be appropriate for comparison in all situations. Furthermore, recent evidence suggests that most data in the DGV overestimate the size of the regions involved because they are dependent primarily on BAC array data, which has a tendency to overestimate the true size of small aberrations [28]. Thus, the most useful CNV databases may be those generated by individual laboratories using identical reference controls and array platforms. Based on our experience, we have constructed a database of abnormal copy number aberrations identified by BAC and oligo aCGH in our laboratory and a database of copy-number variations thought to have no significance. Such databases are essential for understanding the various copy number aberrations identified by microarray analysis.

Genotype-phenotype correlations in a diagnostic setting must address a variety of factors including gene content, potential position effects, aberration size, and inheritance patterns. These factors often present conflicting evidence about the potential clinical significance of a rare alteration. For instance, the size of an abnormality is commonly used as justification for its proposed clinical consequences; however, this association is not always straightforward. High-resolution microarray analysis routinely detects abnormalities smaller than 500 kb that disrupt clinically significant genes and have clear phenotypic impact (Figure 4); conversely, numerous examples of common copy-number variants have been observed that are relatively large but lie in regions with sparse gene content. In addition, although it is generally assumed that *de novo *abnormalities are causative and inherited abnormalities are not, this is not always the case. There are a number of regions of the genome where both inherited and *de novo *copy number alterations have been identified, some of which result in mild phenotypes that may be inherited from parents who have a milder or subclinical, presentation. For example, deletions of distinct regions of 1q21 have been associated with both thrombocytopenia absent radius (TAR) syndrome and a variable phenotype including microcephaly/macrocephaly, developmental delay, cardiac abnormalities, and schizophrenia [29-31], but in many instances aberrations of these regions are inherited from phenotypically normal parents [32]. Another example is the 16p11.2 region associated with a range of cognitive, developmental, and speech delays, behavioral issues, and autism, deletions and duplications of which can be inherited or *de novo *[33-36]. In regions such as these, copy number changes may unmask recessive alleles or work in conjunction with various genetic modifiers, perhaps even other CNVs, to produce a clinical phenotype. Potentially, non-paternity may also confound genotype-phenotype correlation for copy number alterations in these complex regions of the genome. These reasons underscore the need for thorough databases of normal population variants and clinically significant alterations complete with genotype-phenotype correlations. Such databases expedite the process of determining the potential significance of copy-number alterations in a diagnostic setting; aid in the elucidation of new microdeletion/duplication syndromes and new regions of benign copy-number variation; and help reduce the burden of expensive, time-consuming, and difficult follow-up necessitated by the increased number of alterations of unclear clinical significance detected by microarray analysis.

We [19] and others [37] have shown that mosaicism can be detected at low frequencies of chromosomally abnormal cells using BAC-based aCGH; however, the ability of oligo platforms to reliably detect mosaic abnormalities has not yet been well established. Our current assessments demonstrate that aCGH using either BAC or oligo platforms can easily detect mosaicism of 30% or greater for a variety of alterations and that levels as low as 10% can be detected with both platforms under optimal conditions. In addition, our retrospective analysis showed that there is no significant difference between the two types of platforms in the number of mosaic abnormalities detected in a clinical diagnostic setting (p = 0.7066). However, BAC-based arrays may still have a greater ability to detect mosaic abnormalities present at very low levels (less than 20%), perhaps due to the routine use of dye-swap experiments which can be cost-prohibitive with oligo arrays but promote the visual identification of mosaic abnormalities. The sensitivity of the BAC array is demonstrated by the detection in three cases of abnormalities in only 10% of cells during the retrospective study, whereas the lowest level of mosaicism detected by our oligo array was 21% (Table 5). The ability of an aCGH platform to detect mosaic abnormalities also depends largely on the effectiveness of the software used to analyze the data, as low-level mosaic alterations are difficult to identify using only visual inspection (Figure 3). For this reason, it is important to select analysis software which facilitates the identification of mosaic alterations.

These data suggest high-resolution oligo-based aCGH detects a higher proportion of clinically significant abnormalities than BAC-based aCGH. Our results also demonstrate the ability of microarray-based CGH to reliably produce high-yield results in a clinical setting using differing platforms, array designs, and analysis algorithms, supporting the validity of array CGH as a first-tier diagnostic screening tool [38]. Finally, the prevalence of copy number variants of unclear clinical significance detected on both platforms underscores the need for the development of readily accessible diagnostic tools in the form of databases of documented chromosome abnormalities to aid in the interpretation of microarray data.

## Competing interests

NN, BST, BCB, BAB and LGS are employees of Signature Genomic Laboratories, a subsidiary of PerkinElmer.

## Authors' contributions

NN wrote the manuscript; BST analyzed the molecular cytogenetics results; BAB, BCB and LGS designed and coordinated the study. All authors have read and approved the final manuscript.

## References

[B1] Shaffer LG Bejjani BA Torchia B Kirkpatrick S Coppinger J Ballif BC The identification of microdeletion syndromes and other chromosome abnormalities: Cytogenetic methods of the past, new technologies for the future Am J Med Genet C Semin Med Genet 2007 145C 335 345 10.1002/ajmg.c.30152 17910076

[B2] Shevell MI Bejjani BA Srour M Rorem EA Hall N Shaffer LG Array comparative genomic hybridization in global developmental delay Am J Med Genet B Neuropsychiatr Genet 2008 147B 1101 1108 10.1002/ajmg.b.30730 18361433

[B3] Ballif BC Hornor SA Jenkins E Madan-Khetarpal S Surti U Jackson KE Asamoah A Brock PL Gowans GC Conway RL Graham JM Jr Medne L Zackai EH Shaikh TH Geoghegan J Selzer RR Eis PS Bejjani BA Shaffer LG Discovery of a previously unrecognized microdeletion syndrome of 16p11.2-p12.2 Nat Genet 2007 39 1071 1073 10.1038/ng2107 17704777

[B4] Ballif BC Theisen A McDonald-McGinn DM Zackai EH Hersh JH Bejjani BA Shaffer LG Identification of a previously unrecognized microdeletion syndrome of 16q11.2q12.2 Clin Genet 2008 74 469 475 10.1111/j.1399-0004.2008.01094.x 18811697

[B5] Koolen DA Vissers LE Pfundt R de Leeuw N Knight SJ Regan R Kooy RF Reyniers E Romano C Fichera M Schinzel A Baumer A Anderlid BM Schoumans J Knoers NV van Kessel AG Sistermans EA Veltman JA Brunner HG de Vries BB A new chromosome 17q21.31 microdeletion syndrome associated with a common inversion polymorphism Nat Genet 2006 38 999 1001 10.1038/ng1853 16906164

[B6] Sharp AJ Hansen S Selzer RR Cheng Z Regan R Hurst JA Stewart H Price SM Blair E Hennekam RC Fitzpatrick CA Segraves R Richmond TA Guiver C Albertson DG Pinkel D Eis PS Schwartz S Knight SJ Eichler EE Discovery of previously unidentified genomic disorders from the duplication architecture of the human genome Nat Genet 2006 38 1038 1042 10.1038/ng1862 16906162

[B7] Sharp AJ Selzer RR Veltman JA Gimelli S Gimelli G Striano P Coppola A Regan R Price SM Knoers NV Eis PS Brunner HG Hennekam RC Knight SJ de Vries BB Zuffardi O Eichler EE Characterization of a recurrent 15q24 microdeletion syndrome Hum Mol Genet 2007 16 567 572 10.1093/hmg/ddm016 17360722

[B8] Shaw-Smith C Pittman AM Willatt L Martin H Rickman L Gribble S Curley R Cumming S Dunn C Kalaitzopoulos D Porter K Prigmore E Krepischi-Santos AC Varela MC Koiffmann CP Lees AJ Rosenberg C Firth HV de Silva R Carter NP Microdeletion encompassing MAPT at chromosome 17q21.3 is associated with developmental delay and learning disability Nat Genet 2006 38 1032 1037 10.1038/ng1858 16906163

[B9] Vissers LE van Ravenswaaij CM Admiraal R Hurst JA de Vries BB Janssen IM van der Vliet WA Huys EH de Jong PJ Hamel BC Schoenmakers EF Brunner HG Veltman JA van Kessel AG Mutations in a new member of the chromodomain gene family cause CHARGE syndrome Nat Genet 2004 36 955 957 10.1038/ng1407 15300250

[B10] Sharp AJ Emerging themes and new challenges in defining the role of structural variation in human disease Hum Mutat 2009 30 135 144 10.1002/humu.20843 18837009

[B11] Cytrynbaum CS Smith AC Rubin T Weksberg R Advances in overgrowth syndromes: clinical classification to molecular delineation in Sotos syndrome and Beckwith-Wiedemann syndrome Curr Opin Pediatr 2005 17 740 746 10.1097/01.mop.0000187191.74295.97 16282780

[B12] Gropman AL Elsea S Duncan WC Jr Smith AC New developments in Smith-Magenis syndrome (del 17p11.2) Curr Opin Neurol 2007 20 125 134 10.1097/WCO.0b013e3280895dba 17351481

[B13] Ballif BC Theisen A Coppinger J Gowans GC Hersh JH Madan-Khetarpal S Schmidt KR Tervo R Escobar LF Friedrich CA McDonald M Campbell L Ming JE Zackai EH Bejjani BA Shaffer LG Expanding the clinical phenotype of the 3q29 microdeletion syndrome and characterization of the reciprocal microduplication Mol Cytogenet 2008 1 8 10.1186/1755-8166-1-8 18471269 PMC2408925

[B14] Coppinger J McDonald-McGinn D Zackai E Shane K Atkin JF Asamoah A Leland R Weaver DD Lansky-Shafer S Schmidt K Feldman H Cohen W Phalin J Powell B Ballif BC Theisen A Geiger E Haldeman-Englert C Shaikh TH Saitta S Bejjani BA Shaffer LG Identification of familial and de novo microduplications of 22q11.21-q11.23 distal to the 22q11.21 microdeletion syndrome region Hum Mol Genet 2009 18 1377 1383 10.1093/hmg/ddp042 19193630 PMC2664143

[B15] Shaffer LG Theisen A Bejjani BA Ballif BC Aylsworth AS Lim C McDonald M Ellison JW Kostiner D Saitta S Shaikh T The discovery of microdeletion syndromes in the post-genomic era: review of the methodology and characterization of a new 1q41q42 microdeletion syndrome Genet Med 2007 9 607 616 10.1097/GIM.0b013e3181484b49 17873649

[B16] Bejjani BA Saleki R Ballif BC Rorem EA Sundin K Theisen A Kashork CD Shaffer LG Use of targeted array-based CGH for the clinical diagnosis of chromosomal imbalance: Is less more? Am J Med Genet A 2005 134 259 267 15723295 10.1002/ajmg.a.30621

[B17] Bejjani BA Shaffer LG Ballif BC The use of microarray technology for cytogenetics Methods Mol Biol 2010 632 125 139 full_text 20217575 10.1007/978-1-60761-663-4_8

[B18] Traylor RN Fan Z Hudson B Rosenfeld JA Shaffer LG Torchia BS Ballif BC Microdeletion of 6q16.1 encompassing EPHA7 in a child with mild neurological abnormalities and dysmorphic features: case report Mol Cytogenet 2009 2 17 10.1186/1755-8166-2-17 19664229 PMC2731778

[B19] Ballif BC Rorem EA Sundin K Lincicum M Gaskin S Coppinger J Kashork CD Shaffer LG Bejjani BA Detection of low-level mosaicism by array CGH in routine diagnostic specimens Am J Med Genet A 2006 140 2757 2767 17103431 10.1002/ajmg.a.31539

[B20] Lo Nigro C Chong CS Smith AC Dobyns WB Carrozzo R Ledbetter DH Point mutations and an intragenic deletion in LIS1, the lissencephaly causative gene in isolated lissencephaly sequence and Miller-Dieker syndrome Hum Mol Genet 1997 6 157 164 10.1093/hmg/6.2.157 9063735

[B21] Williams SN Locke CJ Braden AL Caldwell KA Caldwell GA Epileptic-like convulsions associated with LIS-1 in the cytoskeletal control of neurotransmitter signaling in Caenorhabditis elegans Hum Mol Genet 2004 13 2043 2059 10.1093/hmg/ddh209 15254012

[B22] Chuang JL Wynn RM Moss CC Song JL Li J Awad N Mandel H Chuang DT Structural and biochemical basis for novel mutations in homozygous Israeli maple syrup urine disease patients: a proposed mechanism for the thiamin-responsive phenotype J Biol Chem 2004 279 17792 17800 10.1074/jbc.M313879200 14742428

[B23] Edelmann L Wasserstein MP Kornreich R Sansaricq C Snyderman SE Diaz GA Maple syrup urine disease: identification and carrier-frequency determination of a novel founder mutation in the Ashkenazi Jewish population Am J Hum Genet 2001 69 863 868 10.1086/323677 11509994 PMC1226071

[B24] Nobukuni Y Mitsubuchi H Akaboshi I Indo Y Endo F Yoshioka A Matsuda I Maple syrup urine disease. Complete defect of the E1 beta subunit of the branched chain alpha-ketoacid dehydrogenase complex due to a deletion of an 11-bp repeat sequence which encodes a mitochondrial targeting leader peptide in a family with the disease J Clin Invest 1991 87 1862 1866 10.1172/JCI115209 2022752 PMC295312

[B25] Poltorak A He X Smirnova I Liu MY Van Huffel C Du X Birdwell D Alejos E Silva M Galanos C Freudenberg M Ricciardi-Castagnoli P Layton B Beutler B Defective LPS signaling in C3H/HeJ and C57BL/10ScCr mice: mutations in Tlr4 gene Science 1998 282 2085 2088 10.1126/science.282.5396.2085 9851930

[B26] Huitema K van den Dikkenberg J Brouwers JF Holthuis JC Identification of a family of animal sphingomyelin synthases EMBO J 2004 23 33 44 10.1038/sj.emboj.7600034 14685263 PMC1271672

[B27] Xu ZH Otterness DM Freimuth RR Carlini EJ Wood TC Mitchell S Moon E Kim UJ Xu JP Siciliano MJ Weinshilboum RM Human 3'-phosphoadenosine 5'-phosphosulfate synthetase 1 (PAPSS1) and PAPSS2: gene cloning, characterization and chromosomal localization Biochem Biophys Res Commun 2000 268 437 444 10.1006/bbrc.2000.2123 10679223

[B28] Bailey JA Kidd JM Eichler EE Human copy number polymorphic genes Cytogenet Genome Res 2008 123 234 243 10.1159/000184713 19287160 PMC2920189

[B29] Brunetti-Pierri N Berg JS Scaglia F Belmont J Bacino CA Sahoo T Lalani SR Graham B Lee B Shinawi M Shen J Kang SH Pursley A Lotze T Kennedy G Lansky-Shafer S Weaver C Roeder ER Grebe TA Arnold GL Hutchison T Reimschisel T Amato S Geragthy MT Innis JW Obersztyn E Nowakowska B Rosengren SS Bader PI Grange DK Recurrent reciprocal 1q21.1 deletions and duplications associated with microcephaly or macrocephaly and developmental and behavioral abnormalities Nat Genet 2008 40 1466 1471 10.1038/ng.279 19029900 PMC2680128

[B30] Stefansson H Rujescu D Cichon S Pietilainen OP Ingason A Steinberg S Fossdal R Sigurdsson E Sigmundsson T Buizer-Voskamp JE Hansen T Jakobsen KD Muglia P Francks C Matthews PM Gylfason A Halldorsson BV Gudbjartsson D Thorgeirsson TE Sigurdsson A Jonasdottir A Bjornsson A Mattiasdottir S Blondal T Haraldsson M Magnusdottir BB Giegling I Moller HJ Hartmann A Shianna KV Large recurrent microdeletions associated with schizophrenia Nature 2008 455 232 236 10.1038/nature07229 18668039 PMC2687075

[B31] Klopocki E Schulze H Strauss G Ott CE Hall J Trotier F Fleischhauer S Greenhalgh L Newbury-Ecob RA Neumann LM Habenicht R Konig R Seemanova E Megarbane A Ropers HH Ullmann R Horn D Mundlos S Complex inheritance pattern resembling autosomal recessive inheritance involving a microdeletion in thrombocytopenia-absent radius syndrome Am J Hum Genet 2007 80 232 240 10.1086/510919 17236129 PMC1785342

[B32] Mefford HC Sharp AJ Baker C Itsara A Jiang Z Buysse K Huang S Maloney VK Crolla JA Baralle D Collins A Mercer C Norga K de Ravel T Devriendt K Bongers EM de Leeuw N Reardon W Gimelli S Bena F Hennekam RC Male A Gaunt L Clayton-Smith J Simonic I Park SM Mehta SG Nik-Zainal S Woods CG Firth HV Recurrent rearrangements of chromosome 1q21.1 and variable pediatric phenotypes N Engl J Med 2008 359 1685 1699 10.1056/NEJMoa0805384 18784092 PMC2703742

[B33] Kumar RA KaraMohamed S Sudi J Conrad DF Brune C Badner JA Gilliam TC Nowak NJ Cook EH Jr Dobyns WB Christian SL Recurrent 16p11.2 microdeletions in autism Hum Mol Genet 2008 17 628 638 10.1093/hmg/ddm376 18156158

[B34] Marshall CR Noor A Vincent JB Lionel AC Feuk L Skaug J Shago M Moessner R Pinto D Ren Y Thiruvahindrapduram B Fiebig A Schreiber S Friedman J Ketelaars CE Vos YJ Ficicioglu C Kirkpatrick S Nicolson R Sloman L Summers A Gibbons CA Teebi A Chitayat D Weksberg R Thompson A Vardy C Crosbie V Luscombe S Baatjes R Structural variation of chromosomes in autism spectrum disorder Am J Hum Genet 2008 82 477 488 10.1016/j.ajhg.2007.12.009 18252227 PMC2426913

[B35] Weiss LA Shen Y Korn JM Arking DE Miller DT Fossdal R Saemundsen E Stefansson H Ferreira MA Green T Platt OS Ruderfer DM Walsh CA Altshuler D Chakravarti A Tanzi RE Stefansson K Santangelo SL Gusella JF Sklar P Wu BL Daly MJ Association between microdeletion and microduplication at 16p11.2 and autism N Engl J Med 2008 358 667 675 10.1056/NEJMoa075974 18184952

[B36] Rosenfeld JA Coppinger J Bejjani BA Girirajan S Eichler EE Shaffer LG Ballif BC Speech Delays and Behavioral Problems are the Predominant Features in Individuals with Developmental Delays and 16p11.2 Microdeletions and Microduplications J Neurodev Disord 2009 0 1 13 10.1007/s11689-009-9037-4 PMC3125720 21731881

[B37] Cheung SW Shaw CA Scott DA Patel A Sahoo T Bacino CA Pursley A Li J Erickson R Gropman AL Miller DT Seashore MR Summers AM Stankiewicz P Chinault AC Lupski JR Beaudet AL Sutton VR Microarray-based CGH detects chromosomal mosaicism not revealed by conventional cytogenetics Am J Med Genet A 2007 143 1679 1686 10.1002/ajmg.a.31740 17607705

[B38] Miller DT Adam MP Aradhya S Biesecker LG Brothman AR Carter NP Church DM Crolla JA Eichler EE Epstein CJ Faucett WA Feuk L Friedman JM Hamosh A Jackson L Kaminsky EB Kok K Krantz ID Kuhn RM Lee C Ostell JM Rosenberg C Scherer SW Spinner NB Stavropoulos DJ Tepperberg JH Thorland EC Vermeesch JR Waggoner DJ Watson MS Consensus statement: chromosomal microarray is a first-tier clinical diagnostic test for individuals with developmental disabilities or congenital anomalies Am J Hum Genet 86 749 764 10.1016/j.ajhg.2010.04.006 20466091 PMC2869000

